# PROMOTING INTERGENERATIONAL EXCHANGES WITHIN RURAL AGE-FRIENDLY COMMUNITIES: A CBPR APPROACH

**DOI:** 10.1093/geroni/igad104.2616

**Published:** 2023-12-21

**Authors:** Victoria Pumilio, Bernard Steinman

**Affiliations:** University of Wyoming, Laramie, Wyoming, United States; University of Wyoming, Laramie, Wyoming, United States

## Abstract

The World Health Organization (WHO) Global Age-Friendly Cities (AFC) guide identifies eight interconnected domains for promoting the well-being, engagement, and participation of older adults in community activities and events. It defines an inclusive society as one that actively encourages older adults to participate in their community’s social, civic, and economic life. Research often prioritizes addressing the physical challenges many older adults face, frequently overlooking societal engagement aspects that promote active aging and inclusion. This research outlines how a Community-Based Participatory Research (CBPR) approach was used to uncover and address the societal stigmas and counterproductive attitudes experienced by older adults in a rural age-friendly designated community. Our CBPR approach is characterized as a process encountered in three stages: (i) conducting a multigenerational survey on age-friendliness, (ii) engaging community members in planning intergenerational opportunities, and (iii) implementing intergenerational engagement activities identified in earlier stages. By participating in methodologies from both CBPR and grounded theory approaches, older adults acquired agency and voice in the research process, thus directly impacting age-friendly outcomes in their communities. Our findings detail the importance of utilizing a CBPR approach when researching core AFC fundamentals that are instrumental in creating inclusive policies and promoting healthful, mutually beneficial intergenerational opportunities.

